# Occurrence and genetic diversity of prophage sequences identified in the genomes of *L. casei* group bacteria

**DOI:** 10.1038/s41598-023-35823-z

**Published:** 2023-05-26

**Authors:** Piotr Jarocki, Elwira Komoń-Janczara, Agata Młodzińska, Jan Sadurski, Kinga Kołodzińska, Łukasz Łaczmański, Jacek Panek, Magdalena Frąc

**Affiliations:** 1 Department of Biotechnology, Microbiology and Human Nutrition, University of Life Sciences in Lublin, 8 Skromna St., 20-704 Lublin, Poland; 2 Bioidea Company, Warsaw, Poland; 3 Laboratory of Genomics and Bioinformatics, Hirszfeld Institute of Immunology and Experimental Therapy, Polish Academy of Sciences, Wrocław, Poland; 4 Department of Soil and Plant System, Laboratory of Molecular and Environmental Microbiology, Institute of Agrophysics, Polish Academy of Sciences, Lublin, Poland

**Keywords:** Microbiology, Microbial genetics, Phage biology, Virology

## Abstract

It is widely believed that microorganisms belonging to *L. casei* group can have positive effects on the human body. Therefore, these bacteria are used in many industrial processes, including the production of dietary supplements and probiotic preparations. When using live microorganisms in technological processes, it is important to use those without phage sequences within their genomes that can ultimately lead to lysis of the bacteria. It has been shown that many prophages have a benign nature, meaning that they don’t directly lead to lysis or inhibit microbial growth. Moreover, the presence of phage sequences in the genomes of these bacteria increases their genetic diversity, which may contribute to easier colonization of new ecological niches. In the 439 analyzed genomes of the *L. casei* group, 1509 sequences of prophage origin were detected. The average length of intact prophage sequences analyzed was just under 36 kb. GC content of tested sequences was similar for all analyzed species (44.6 ± 0.9%). Analyzing the protein coding sequences collectively, it was found that there was an average of 44 putative ORFs per genome, while the ORF density of all phage genomes varied from 0.5 to 2.1. The average nucleotide identity calculated on sequence alignments for analyzed sequences was 32.7%. Of the 56 *L. casei* strains used in the next part of the study, 32 did not show culture growth above the OD600 value of 0.5, even at a mitomycin C concentration of 0.25 μg/ml. Primers used for this study allowed for the detection of prophage sequences for over 90% of tested bacterial strains. Finally, prophages of selected strains were induced using mitomycin C, phage particles were isolated and then genomes of viruses obtained were sequenced and analyzed.

## Introduction

Bacteriophages, or phages for short, are viruses that infect bacterial cells and are considered the black matter of biodiversity. It has been proposed that they not only influence the number of microorganisms but also indirectly affect the functioning of entire ecosystems^1,2^. Due to the very high variability of viruses, an accurate estimation of the number of bacteriophages in the environment is a big challenge for the scientific community. Conservative estimates indicate that the number of free phage particles is 10 times higher compared to the number of bacterial cells. This makes bacteriophages the most abundant biological structures found in nature^3^. The consequences of high genetic diversity are diverse morphology and different types of life cycles. In addition to the well-studied lytic cycle leading to the complete lysis of bacterial cultures, other types of infections have been described that ensure the survival of bacteriophages and do not cause the death of bacterial host cells. These include widespread lysogeny, pseudolysogeny and chronic infection^3,4^. It should be noted that, compared to lytic phages, our understanding of the physiological significance of the non-lytic forms of bacterial virus forms is still insufficient. Moreover, by analyzing the astonishing genetic diversity of bacteriophages, it can be assumed that the previously described interactions between phages and bacterial cells are not the only ways in which the bacteriophage life cycle is completed. It is likely that many aspects of this matter remain undiscovered.

Lysogeny is a common phenomenon in bacteria of the genus *Lactobacillus,* including those belonging to the *L. casei* group^5–8^. Bacteria of this group, as a result of reclassification of the genus *Lactobacillus,* now belong to the genus *Lacticaseibacillus*, which includes 17 species including *Lacticaseibacillus casei*, *Lacticaseibacillus paracasei* and *Lacticaseibacillus rhamnosus,* traditionally considered as the *L. casei group*. These bacteria can be isolated from a diverse range of environments, such as traditional fermented products (dairy, cereal, meat), humans and animals (oral cavity, vagina, gastrointestinal tract), invertebrate hosts, sewage and clinical sources^9,10^. It is worth noting that several biochemical and physiological characteristics predispose some strains of the *L. casei* group to be used in the production of dietary supplements and probiotic drugs—the effectiveness of which in the prevention and treatment of certain diseases has been previously scientifically documented^11^.

Considering that these bacteria are used in many industrial processes, the potential for induction and lysis would bring about negative consequences for the industries in which they are used^8^. On the other hand, many studies indicate that lysogenic phages have a benign nature, and do not cause complete lysis of bacterial cultures even when induced^5,9,10^. The process of stimulating the life cycle itself, resulting in the formation of phage particles, can be caused by environmental factors, such as changes in pH and temperature, poor nutrient availability, or exposure to reactive oxygen species and antibiotics^4,11,12^. Prophage induction can also be spontaneous. During spontaneous prophage induction (SPI), only a small subpopulation of cells undergo induction and release mature bacteriophages. This process can be caused by extracellular factors or can result from certain intracellular processes^13–15^.

Interestingly, there appears to be physiological importance of the co-existence of phages and bacterium. Some researchers suggest that this phenomenon may bring potential benefits for the bacterial host, by introducing resistance to other phages or by enhancing the capability of the bacteria to form biofilms, and therefore increase the survivability of microorganisms in their natural ecological niche. Furthermore, the occurrence of prophage sequences in bacterial genomes has also been linked to the potential pathogenicity of microorganisms, which seems particularly relevant to microorganisms living in the human body^16–18^.

In this work, the occurrence and genetic diversity of prophage sequences presented in the genomes of *L. casei* group bacteria were studied using various bioinformatic and molecular tools. Following this data, the ecological and physiological significance of this phenomenon was discussed.

## Results

### Occurrence of prophage sequences in *L. casei* group genomes

The first stage of the analysis involved the identification of prophage-like sequences in 439 genomic sequences of bacterial strains belonging to the following 5 species: *L. casei* (27), *L. paracasei* (200), *L. rhamnosus* (204), *L. zeae* (6) and *L. chiayiensis* (2). In the analyzed genomes, 1509 sequences showing prophage origin (including incomplete, questionable and intact sequences) were detected using the Phaster software (Table 1, Additional file 1 and Additional file 2). For the 5 genomic sequences, no positive results were obtained. Only the intact prophage sequences were used for further analysis—duplicated (in the case of strains whose genomes were sequenced several times), incomplete and questionable sequences were removed. The basic characteristics of the rejected sequences (incomplete and questionable sequences) are shown in Additional file 3. In the following stages, 422 intact sequences that scored above 90 in the Phaster software were analyzed. Among the intact sequences, 24 were from the genomes of *L. casei*, 232 from *L. paracasei*, 162 from *L. rhamnosus*, 4 from *L. zeae*, and none from *L. chiayiensis*. The classification of these species is currently a contentious issue, with some studies indicating that *L. zeae* strains should be included in the *L. casei* species^10^. It is also worth noting that larger amounts of intact prophage sequences were detected in strains belonging to *L. casei* and *L. paracasei* species—as many as 30 genomic sequences contained between 3 and 5 prophages. For *L. rhamnosus*, a maximum of 2 prophage sequences were detected in a single genome (Additional file 2).

**Table 1:** Characterization of phage sequences identified in *L. casei* group bacteria genomes.

Species	No. of intact sequences	No. of questionable sequences	No. of incomplete sequences
*L. casei*	25	18	54
*L. paracasei*	247	272	319
*L. rhamnosus*	172	199	173
*L. zeae*	4	9	11
*L. chiayiensis*	0	3	3
Summary	448	501	560

**Figure 1:**
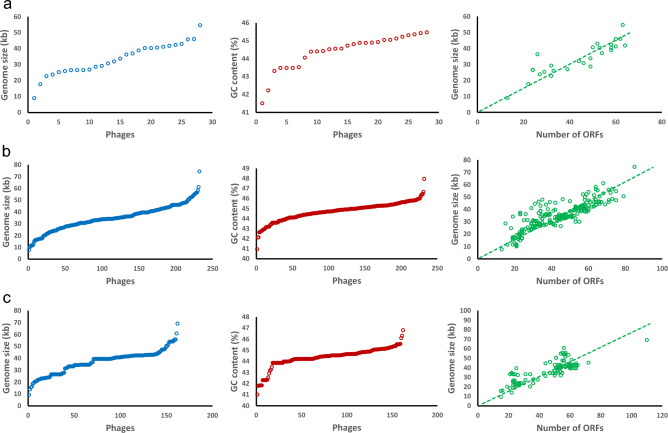
Characterization of detected intact prophage genomes in terms of size (kb), percentage of GC, and the number of identified ORFs for each species: (**a**) *Lacticaseibacillus casei*, (**b**) *Lacticaseibacillus paracasei*, and (**c**) *Lacticaseibacillus rhamnosus.*

The average length of the prophage sequences analyzed was just under 36 kb (35.6 ± 10.3 kb). The length of the prophage regions within the genomes of the *L. casei* strains ranged from 9 to 54.7 kb (mean 33.7 ± 10 kb) (Fig. 1a); from 7.7 to 74.4 kb (mean 34.9 ± 10.6 kb) for *L. paracasei* (Fig. 1b); and from 9.3 to 69.2 kb (mean 37 ± 9.8 kb) for sequences extracted from *L. rhamnosus* genomes (Fig. 1c). As for the GC content ratio for the sequences studied, it was at a similar level for all analyzed species at an average of 44.57 ± 0.91 (Fig. 1 and Additional file 4). Detailed information regarding individual prophage sequences is provided in Additional files 2 and 5.

The number of ORFs detected within the prophage sequences was determined by the length of the prophage. For the shortest sequence (7.7 kb) detected for *L. paracasei* TD 62 (CP044363.1), 13 ORFs were identified. An identical number of ORFs were observed in a prophage derived from the sequence of strain DS13_13 (QAZE01000062.1) belonging to the *L. casei* species. In this case, the length of the prophage sequence was slightly longer, reaching 9 kb. For the longest sequences detected in the genomes of *L. casei* L.cR4 (JAAQWB010000003.1), *L. paracasei* JS1 (QHHM01000001.1) and *L. rhamnosus* IDCC 3201 (CP045531.1); 63, 85 and 110 protein-coding genes were identified, respectively (Additional file 5). Analyzing the protein-coding sequences collectively, it was found that there were more than 44 putative ORFs per genome on average, and the ORF density of all phage genomes varied from 0.52 to 2.08 ORFs/kb. A similar average ORF density value ranging from 1.23 to 1.26 ORFs/kb was obtained between the analyzed species.

### Assembling phage genomes into clusters

**Figure 2:**
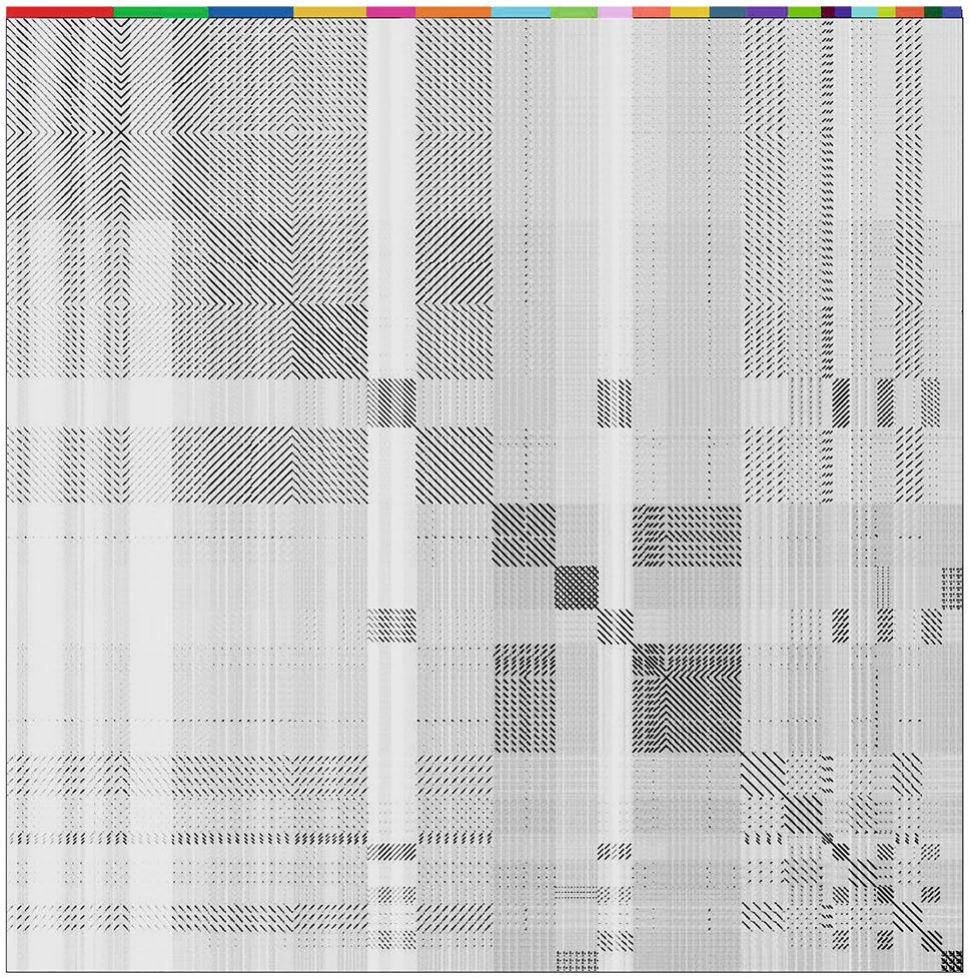
Whole-genome dot plot comparison of 129 prophage sequences classified into 21 clusters. All sequences were concatenated into a single sequence and plotted against itself. The results were visualized by Gepard.

The classification of phage sequences into clusters was carried out in accordance with previous work by Ha et al.^19^. The resulting dot plot matrix shows the 129 sequences classified into 21 phage clusters, showing nucleotide sequence similarity of more than 45% (Fig. 2, Table 2 and Additional file 6). The remaining sequences were referred to as singletons. The largest cluster was cluster 1, which included 14 sequences, while only 3 bacteriophage sequences were grouped in clusters 15 through 21 each. When analyzing the distribution of prophage sequences, in terms of the bacterial host species from which the sequences originated, it was found that only 2 clusters contained prophages from more than one species. Eleven clusters contained sequences derived from *L. rhamnosus*, 7 clusters included sequences obtained from *L. paracasei* and 1 cluster grouped 3 sequences obtained for *L. casei* strains. Details of the species affiliation of the individual bacterial strains from which the analyzed prophage sequences were derived and the source of their isolation are presented in Additional file 5. Analyzing the prophage sequences from a clustering perspective, it is important to note the wide variation in the average length of the sequences studied, ranging from 18.2 kb for cluster 15 to 54.1 kb for cluster 13. The average GC content of each cluster ranged from 43.5 to 45.8%, with only the sequences contained in clusters 9 and 20 having a slightly lower average GC percentage value of 42.3% and 41.8%, respectively. As for the average number of protein-coding sequences in each cluster, considerable variation was observed: from 21.3 in cluster 15, to more than 65 in clusters 14 and 17. A large variation in the average number of coding sequences per kilobase was also observed, ranging from 0.64 to 1.45 ORFs/kb. In clusters 5, 9, 16, 20 (*L. rhamnosus*), 18 (*L. casei*) and 21 (*L. paracasei*) the average ORFs/kb value was less than 1. For the remaining clusters, a value above 1 was obtained, while the highest number of identified coding sequences per kilobase was observed for sequences clustered in clusters 2, 3, 6, 11, 14 and 17. For these sequences, average values above 1.4 ORFs/kb were obtained.

**Table 2:** Characterization and clustering of detected prophage sequences.

Cluster	No. of sequences	Average length	GC content	Average no. of ORFs	Average no. of ORFs per kb	Hosts
1	14	40.58 ± 2.50	44.14 ± 0.17	54.14 ± 5.93	1.34 ± 0.14	*L. rhamnosus* (14)
2	11	39.50 ± 0.00	44.22 ± 0.00	55.27 ± 4.67	1.40 ± 0.12	*L. rhamnosus* (11)
3	10	41.64 ± 2.58	44.49 ± 0.13	62.10 ± 3.03	1.49 ± 0.04	*L. rhamnosus* (10)
4	10	37.50 ± 3.12	44.73 ± 0.10	50.30 ± 3.23	1.34 ± 0.03	*L. rhamnosus* (9)
5	9	26.50 ± 0.00	43.86 ± 0.00	23.11 ± 0.33	0.87 ± 0.01	*L. rhamnosus* (9)
6	9	42.33 ± 0.10	44.52 ± 0.09	60.55 ± 0.88	1.43 ± 0.02	*L. rhamnosus* (9)
7	8	38.89 ± 1.89	45.23 ± .0.14	51.88 ± 2.09	1.33 ± 0.03	*L. paracasei* (8)
8	7	30.71 ± 0.04	43.57 ± 0.06	30.86 ± 0.69	1.01 ± 0.02	*L. paracasei* (7)
9	5	34.52 ± 0.18	42.32 ± 0.01	22.00 ± 0.00	0.64 ± 0.00	*L. rhamnosus* (5)
10	6	27.67 ± 5.20	45.79 ± 0.53	36.66 ± 9.43	1.31 ± 0.11	*L. paracasei* (5) *L. casei* (1)
11	5	34.12 ± 0.04	45.61 ± 0.06	48.20 ± 1.09	1.41 ± 0.03	*L. paracasei* (5)
12	6	33.83 ± 1.71	45.33 ± 0.31	44.50 ± 2.43	1.32 ± 0.07	*L. paracasei* (6)
13	4	54.10 ± 0.00	45.12 ± 0.00	56.50 ± 0.58	1.04 ± 0.01	*L. rhamnosus* (4)
14	4	46.00 ± 0.00	44.91 ± 0.01	65.5 ± 1.73	1.42 ± 0.04	*L. paracasei* (4)
15	3	18.23 ± 1.38	44.89 ± 0.30	21.33 ± 1.15	1.17 ± 0.07	*L. paracasei* (2) *L. zeae* (1)
16	3	26.50 ± 0.00	43.86 ± 0.00	24.00 ± 0.00	0.91 ± 0.00	*L. rhamnosus* (3)
17	3	46.07 ± 0.01	44.13 ± 0.01	65.33 ± 1.16	1.42 ± 0.02	*L. paracasei* (3)
18	3	26.60 ± 0.00	43.49 ± 0.00	24.00 ± 0.00	0.90 ± 0.00	*L. casei* (3)
19	3	47.33 ± 0.06	45.11 ± 0.00	56.00 ± 0.00	1.18 ± 0.00	*L. rhamnosus* (3)
20	3	33.20 ± 0.00	41.81 ± 0.02	23.00 ± 1.73	0.69 ± 0.05	*L. rhamnosus* (3)
21	3	36.20 ± 0.00	44.11 ± 0.00	23.00 ± 0.00	0.64 ± 0.00	*L. paracasei* (3)

### Screening of *L. casei* group using a PCR-based method designed for prophage identification

The second part of the study used 56 strains of the *L. casei* group, obtained from two international collections of microorganisms (BCCM and JCM). The strains studied came from different geographic locations and diverse ecological niches (Table 3). In the first phase, the species affiliation of the tested strains was analyzed using procedures described in earlier work (multiplex PCR and species-specific PCR)^20^. Interestingly, of the 56 strains surveyed, the original species affiliation was not confirmed in 7 cases. For 2 strains; *L. casei* JCM 8677 and *L. rhamnosus* LMG 10775, no positive results were obtained using PCR-based methods. LC–MS/MS analysis showed that the strains belonged to the *L. crispatus* and *L. curvatus* species. For the remaining 5 strains, isolates JCM 2120, JCM 20024, JCM 8648 and JCM 20304 (originally *L. casei*) were classified as *L. paracasei*, while strain JCM 8608 (also originally *L. casei*) was classified as *L. rhamnosus* (Table 3). In a preliminary study described in an earlier paper by Jarocki et al.^20^, interspecific differentiation of the tested strains was also analyzed using methods such as amplified fragment length polymorphism (AFLP) and Rep-PCR. The obtained results made it possible to observe fundamental differences in the genetic profiles, both at the species level and between individual strains. The observations made are likely due to the diverse origins of the studied microorganisms as well as the presence of repetitive sequences and mobile fragments such as prophages in the genomes.

**Table 3:** PCR assay for prophage identification in the genomes of *L. casei* group strains used in this study. Prophage detection was performed using 9 primer sets described previously by Zaburlin et al.^7^. Bacterial growth in the presence of various concentrations of mitomycin C for all tested strains was also presented.

Species	Strain	Origin	Group 1 *iA2-like* prophages			Group 2 CL1, CL2, i*Lp*84, i*Lp*1308		Group 3 A2, Lrm1, PL-1, J-1, AT3				Growth with mitomycin C [µg/ml]		
			Pair 1	Pair 2	Pair 3	Pair 4	Pair 5	Pair 6	Pair 7	Pair 8	Pair 9	0.25	0.50	1.00
*L. casei*	LMG 6904	Cheese	−^1^	#^2^	#	#	#	+^3^	#	+	#	~^4^	~	~
*L. casei*	LMG 23516	Human faeces (Italy)	−	#	#	#	#	+	#	+	#	~	~	~
*L. casei*	LMG 24099	Human, blood (Belgium)	−	#	#	#	#	+	#	+	#	**^6^	~	~
*L. casei*	LMG 24102	Heart valve tissue (Antwerpen, Belgium)	−	#	#	#	#	+	#	+	#	***^7^	~	~
*L. casei*	JCM 8129	Milk products	−	#	−	#	#	+	#	+	#	~	~	~
*L. casei *(*zeae*)	LMG 17315	Corn steep liquor	−	#	#	#	#	+	+#	+	+#	~	~	~
*L. paracasei*	JCM 2120	−	+	+	−	#	−	+	+	+	+	~	~	~
*L. paracasei*	JCM 8648	Fermented cane molasses (Thailand)	−	−	#	+	+	#	#	−	#	~	~	~
*L. paracasei*	JCM 20304	–	+	+	−	#	#	+	#	+	#	~	~	~
*L. paracasei*	JCM 20024	Cheese	−	−	−	+	+	+	+#	−	+#	*^5^	*	*
*L. paracasei*	LMG 13087	−	−	−	#	+#	#	+	#	+	#	~	~	~
*L. paracasei*	LMG 9193	−	+	+	#	+#	#	+	+	+	+	**	*	~
*L. paracasei*	LMG 9438	Child, saliva	−	#	#	+#	+	−	#	−	#	*	~	~
*L. paracasei*	LMG 11459	Dental caries (UK)	−	−	−	+	#	+	+	+	+	~	~	~
*L. paracasei*	LMG 11961	Port wine (Portugal)	−	−	−	+	+	+	+#	+	+#	~	~	~
*L. paracasei*	LMG 12164	Homemade hard cheese (Livno, Yugoslavia)	+	+	−	#	#	+	+#	+	+#	~	~	~
*L. paracasei*	LMG 19719	Blood culture (Denmark)	−	−	−	#	#	+	#	+	#	~	~	~
*L. paracasei*	JCM 1163	Beer	−	#	−	−	#	+	+#	+	+#	~	~	~
*L. paracasei subsp. tolerans*	LMG 9191	Pasteurized milk	−	#	#	+	+	+	#	+	#	~	~	~
*L. paracasei subsp. tolerans*	JCM 1172	Pasteurized milk	−	#	#	+	+	+	#	+	#	~	~	~
*L. paracasei*	LMG 7955	−	−	−	#	#	#	+	+#	+	+#	~	~	~
*L. paracasei*	LMG 8157	– (Göteborg, Sweden)	+	+	−	+	#	+	+	+	+	*	~	~
*L. paracasei*	JCM 20315	–	−	−	#	+	+	+	+#	+	+#	~	~	~
*L. paracasei*	JCM 1109	Human intestine	−	#	−	−	#	+	+	+	+	~	~	~
*L. paracasei*	JCM 8133	Milk products	+	+	−	+	+	+	#	+	#	~	~	~
*L. rhamnosus*	LMG 6400	–	−	−	#	#	#	#	#	#	#	~	~	~
*L. rhamnosus*	JCM 8608	Fermented cane molasses (Thailand)	−	#	#	#	−	#	#	−	#	**	~	~
*L. rhamnosus*	LMG 8153	Healthy adult female, urethra (Canada)	−	−	#	#	#	#	#	−	#	*	~	~
*L. rhamnosus*	LMG 10768	Blood (Sweden)	−	#	−	#	#	+	+#	+	+#	***	~	~
*L. rhamnosus*	LMG 10772	Wine (Sweden)	−	#	−	+	+#	#	#	−	#	**	*	~
*L. rhamnosus*	LMG 12166	Homemade hard cheese (Surcin, Yugoslavia)	−	−	−	#	+#	#	#	−	#	*	~	~
*L. rhamnosus*	LMG 18030	Zabady, yoghurt (Alexandria, Egypt)	−	−	−	#	#	−	#	#	#	~	~	~
*L. rhamnosus*	LMG 23304	Human, feces (Belgium)	−	#	−	#	#	+	#	+	#	**	*	~
*L. rhamnosus*	LMG 23536	Healthy human, adult, faeces (Kuopio, Finland)	−	−	#	#	+	−	#	−	−	~	~	~
*L. rhamnosus*	LMG 23550	Human with endocarditis, blood (UK)	−	−	#	#	+	+	+#	+	+#	~	~	~
*L. rhamnosus*	LMG 25881	Dairy product (China)	−	#	−	+	+#	−	#	−	#	**	*	~
*L. rhamnosus*	LMG 10770	Bowel drain (Uppsala, Sweden)	−	−	#	#	#	#	+	#	+	**	~	~
*L. rhamnosus*	LMG 10773	Sputum (Stockholm, Sweden)	−	−	#	+	+#	#	+	#	+	*	~	~
*L. curvatus*	LMG 10775	Hip punction (Huddinge, Sweden)	−	−	−	#	#	−	#	#	−	*	*	~
*L. rhamnosus*	LMG 10776	Pleura (Skövde, Sweden)	−	−	#	+	+#	+	+	+	+	**	~	~
*L. rhamnosus*	LMG 18020	Mish cheese (Delta Area, Egypt)	−	−	#	#	+	#	#	−	#	***	***	**
*L. rhamnosus*	LMG 18028	Laban Rayeb (Cairo, Egypt)	−	−	#	#	+#	+	#	+	#	**	**	~
*L. rhamnosus*	LMG 19716	Blood culture (Denmark)	−	−	#	#	#	−	#	−	#	**	*	~
*L. rhamnosus*	LMG 19717	Blood, terminal ileitis (Denmark)	−	#	−	#	#	+	#	+	#	~	~	~
*L. rhamnosus*	LMG 23291	Human, abscess (Belgium)	−	−	#	#	#	+	#	+	#	~	~	~
*L. rhamnosus*	LMG 23296	Human, bronchoalveolar lavage fluid (Belgium)	−	−	#	#	#	+	#	+	#	~	~	~
*L. rhamnosus*	LMG 23285	Human, pus diverticulitis (Belgium)	−	#	−	#	#	+	#	+	#	*	*	~
*L. rhamnosus*	LMG 23278	Human, pleural fluid (Belgium)	−	−	#	−	#	+	#	+	#	~	~	~
*L. rhamnosus*	LMG 23227	Human, blood (Belgium)	−	−	−	#	#	+	#	+	#	~	~	~
*L. rhamnosus*	LMG 23327	Human, blood	−	#	−	#	#	+	#	+	#	*	~	~
*L. rhamnosus*	LMG 23527	Healthy human, adult, faeces (Kuopio, Finland)	−	−	#	#	#	+	#	+	#	**	**	~
*L. rhamnosus*	LMG 23551	Human with endocarditis, blood (UK)	−	−	−	#	+#	+	#	+	#	*	*	~
*L. rhamnosus*	LMG 23667	5-day-old child, faeces (Sweden)	−	#	#	#	#	#	+	#	+	~	~	~
*L. rhamnosus*	LMG 25626	Human, intestine (Belgium)	−	−	#	#	+	−	#	−	−	~	~	~
*L. rhamnosus*	LMG 27305	Grafted milk (Italy)	−	−	#	#	#	+	+	+	+	~	~	~
*L. crispatus*	JCM 8677	Adult	−	−	−	#	#	−	−	−	−	~	~	~

^1^No PCR product/no bacterial growth.

^2^Presence of non-specific PCR product(s).

^3^Specific PCR product of expected length.

^4^Bacterial culture growth in the range of OD_600_ values below 0.5

^5^Bacterial culture growth in the range of OD_600_ values from 0.5 to 1.0

^6^Bacterial culture growth in the range of OD_600_ values from 1.0 to 1.5

^7^Bacterial culture growth in the range of OD_600_ values above 1.5

The pool of strains was then tested for growth in the presence of various concentrations of mitomycin C. A noticeable decrease in the growth curve of the bacteria may suggest the occurrence of complete or partial lysis. On the other hand, the inhibition of bacterial growth may be related to the toxic effects of mitomycin C itself on microbial cells. Table 3 shows the results of measuring the OD600 of bacterial cultures supplemented with different concentrations of mitomycin C. Among the 56 isolates, 32 did not show culture growth above the OD600 value of 0.5, even at a concentration of 0.25 µg/ml mitomycin C. At the lowest tested concentration of mitomycin C, 2 strains of *L. casei*, 3 strains of *L. paracasei* and 17 strains of *L. rhamnosus* showed culture growth. At a concentration of 0.5 µg/ml, bacterial growth was observed for 11 bacterial strains from the *L. casei* group (1 each of *L. casei* and *L. paracasei* and 9 belonging to *L. rhamnosus*), while a concentration of 1 µg/ml was tolerated by only 2 bacterial strains; *L. paracasei* JCM 20024 and *L. rhamnosus* LMG 18020. Noteworthy here is the lower sensitivity to mitomycin C of *L. rhamnosus* strains, among which strain LMG 18020 showed by far the highest resistance.

In an earlier paper, Zaburlin et al. developed a set of primers for detecting prophages and prophage remnants in *L. casei* group genomes^7^. Based on the results, it was possible to classify the identified prophage-like sequences into 3 groups: group 1 (*iA2-like* prophages), group 2 (CL1, CL2, i*Lp*84 and i*Lp*1308-like prophages), and group 3 (phage A2, Lrm1, PL-1, J-1 and AT3-like prophages). In the present study, group 1 sequences were detected for 6 strains (Table 3). The prophage sequences were detected using primer pairs 1 and 2, which were designed based on genes encoding HNH endonuclease and terminase large subunit (TLS). The detected prophages were from strains belonging to the *L. paracasei* species. Using a third pair of primers for none of the 56 strains tested, a specific PCR product of 668 bp could be obtained. In this case, 19 strains of *L. rhamnosus* generated a single PCR product of about 750 bp in length.

For the second group of prophages (similar to phages CL1, CL2, i*Lp*1308 and i*Lp*84), primers were designed based on the gene sequences encoding TLS and portal proteins. Using these primer sets, positive results were obtained for 23 strains (16 with pair 4 and 19 with pair 5 primers), of which 12 belonged to *L. paracasei* and 11 belonged to *L. rhamnosus*. Among these strains, 3 were also positive in an earlier test that identified *iA2-like* prophages (group 1). Four sets of primers were used to detect the last group of prophages, similar to bacteriophages A2, Lrm1, PL-1, J-1 and AT3. A third group of prophage sequences was identified for 41 strains belonging to the 3 species tested, including 24 strains in which prophages from groups 1 and 2 were not detected. Using primer pairs 6 and 8, it was possible to detect 38 and 37 prophage sequences, respectively, indicating the high conservation between the amplified fragments (Table 3).

In summary, phage sequences were detected in 48 of the 56 bacterial strains using the described primer sets. Two of the remaining 8 strains were non-*L. casei* control strains. The remaining 6 isolates for which negative results were obtained belonged to the *L. rhamnosus* species. The genomes of these strains lack prophage-like sequences or, due to the very high diversity of prophages, the procedure used was not sufficient to detect all such sequences.

### Sequencing and characterization of phage genomes obtained by induction of selected *L. casei* bacterial strains

The next part of the study used 12 bacterial strains which were selected based on previous research results. In order to induce the previously detected prophages, the test strains were cultured in MRS medium supplemented with mitomycin C. Harvested phage particles were then purified and genetic material was isolated. Sequencing was performed using Illumina's MiSeq system, and the resulting contig sequences were analyzed with Phaster software to confirm the presence of phage sequences. The results were consistent with the analysis performed by PCR reaction—at least 1 phage sequence was identified for each strain (Tables 3 and 4).

**Table 4:** Basic genomic features of temperate phages induced from selected strains belong to the *L. casei* group.

Phage name	Host	Completeness (score)	Average coverage	Genome size (bp)	GC%	No. of ORFs	Similar phage (cover/identity)	Accession number
C3.1	*L. casei* LMG 24099	Intact (93)	130.52	40,498	44.43	55	T25 (66/91)	OP676228
C4.1	*L. casei* LMG 24102	Intact (93)	115.45	40,496	44.43	55	T25 (66/91)	OP807341
P2.4	*L. paracasei* LMG 9193	Intact (150)	97.49	31,327	46.25	44	A2 (73/97)	OP807342
P7.1	*L. paracasei* LMG 19719	Intact (150)	131.75	41,308	44.43	55	JNU_P10 (24/90)	OP807343
R3.1	*L. rhamnosus* LMG 10768	Questionable (86)	108.06	41,371	44.61	58	BH1 (66/93)	OP807344
R3.3	*L. rhamnosus* LMG 10768	Intact (150)	55.73	38,073	45.75	58	PLE3 (15/95)	OP807345
R9.1	*L. rhamnosus* LMG 23550	Questionable (80)	143.69	43,914	45.08	64	JNU_P10 (50/90)	OP807346
R9.2	*L. rhamnosus* LMG 23550	Incomplete (20)	28.17	16,704	43.55	30	JNU_P9 (54/94)	OP869843
R9.3	*L. rhamnosus* LMG 23550	Intact (120)	29.68	22,598	44.26	26	Lc-Nu (88/93)	OP869844
R10.1	*L. rhamnosus* LMG 25881	Questionable (80)	168.77	37,715	45.10	58	JNU_P10 (54/87)	OP869845
R18.1	*L. rhamnosus* LMG 19717	Intact (120)	131.91	41,877	44.49	53	BH1 (61/93)	OP869846
R23.1	*L. rhamnosus* LMG 23277	Incomplete (20)	332.87	14,518	43.64	22	–	OP869847
R23.9	*L. rhamnosus* LMG 23277	Questionable (86)	114.19	41,598	45.18	54	BH1 (66/91)	OP869848
R24.1	*L. rhamnosus* LMG 23327	Questionable (81)	91.16	40,600	44.31	59	JNU_P9 (68/93)	OP869849
R24.2	*L. rhamnosus* LMG 23327	Questionable (77)	161.98	42,770	44.70	67	JNU_P10 (53/90)	OP869850
R26.1	*L. rhamnosus* LMG 23551	Incomplete (30)	82.35	15,427	44.58	17	LJ (92/92)	OP869851
R26.9	*L. rhamnosus* LMG 23551	Incomplete (60)	35.19	13,665	46.12	16	JNU_P10 (83/90)	OP869852
R26.14	*L. rhamnosus* LMG 23551	Incomplete (30)	58.92	11,745	43.00	17	T25 (57/91)	OP869853
R26.26	*L. rhamnosus* LMG 23551	Incomplete (20)	26.24	16,938	43.31	35	JNU_P10 (42/96)	OP869854
R29.1	*L. rhamnosus* LMG 27305	Questionable (86)	207.94	41,353	44.62	59	BH1 (66/93)	OP869855

Analysis with the Phaster program showed that 7 intact sequences, 7 questionable sequences and 6 incomplete sequences were identified in the obtained contigs (Table 4). In the case of intact phages, their length ranged from 22,598 bp (contig 9.3 obtained for *L. rhamnosus* LMG 23550) to 41,877 bp (phage sequence extracted from *L. rhamnosus* strain LMG 19717). The GC percentage for these phages ranged from 44.26 to 46.25%, while the number of identified ORFs ranged from 26 to 58. For the questionable type of sequences, for which scores in the range of 77 to 86 were obtained using Phaster, similar lengths were obtained (from about 37,000 bp to just under 44,000 bp). Comparative analysis of these sequences against bioinformatics databases, containing genome sequences of bacteriophages and *Lacticaseibacillus,* suggests that the phages analyzed here contain fully functional sequences, while the relatively low scores obtained in Phaster may be due to the very high genetic variability of the phages described for the bacterial species studied. In the case of 6 incomplete sequences whose lengths were much shorter (11,000–17,000 bp), it can be concluded that the analyzed contigs contained only fragments of phage-like sequences. On the other hand, a careful analysis of these sequences showed that in the case of contig 9.2 (derived from *L. rhamnosus* strain LMG 23550), the identified sequence is probably part of the phage detected in contig 9.3. A similar situation also applies to the 2 contigs obtained for *L. rhamnosus* LMG 23277 (23.1 and 23.9). For another *L. rhamnosus* strain, LMG 23277, as many as four contigs containing phage sequences (R26.1, R26.9, R26.14 and R26.26) were obtained. It can be speculated that in this case, both the high genetic diversity of the phages studied, and the relatively low coverage obtained for some contigs, may have made it much more difficult to assemble the sequences obtained by NGS sequencing.

The phage sequences obtained were then compared to the genomes of previously described bacteriophage and prophage sequences present in the genomes of *L. casei* bacteria. This confirmed previous analysis showing extreme variation in the phage sequences for the species studied. Interestingly, very similar sequences of phage origin have also been identified in sequencing data from human metagenome samples^21^.

In the case of the C3.1 and C4.1 phage sequences, which showed very high similarity between them, a 91% match was observed with the T25 phage sequence described for the *L. paracasei* species. However, similarity at this level covered only 66% of the sequences. On the other hand, an identical prophage sequence was identified in the genome of an *L. casei* bacterium (strain BIO5773). For the next two phages; P2.4 and P7.1, obtained from *L. paracasei* strains, the highest similarity was obtained with previously characterized phages iA2 and JNU_P10. In both cases, the sequence similarity was above 90%, however, coverage of similar sequences was 73% and 24%, respectively. Genome sequences of viruses isolated from *L. rhamnosus* cultures showed similarity with bacteriophages BH1, PLE3, JNU_P10, JNU_P9, Lc-Nu, LJ and T25. It should be noted that the detected sequence match concerned only fragments of the above phages. For phage R23.1, no similarity could be identified with previously described phages described for the *L. casei* group. Despite this, a very similar prophage sequence was observed in the genome of *L. rhamnosus* strain PMC203. The same or very similar prophage sequences in the *L. rhamnosus* genomes were also detected for phages R3.1, R10.1, 23.9, 24.2 and 29.1 (Table 4).

### Genome structure of *L. casei* group bacteriophages

In the next step, the genomes of bacteriophages were analyzed for the presence of protein-coding sequences. A detailed characterization of the identified ORFs as well as their protein products is presented in Additional file 7.

The *L. casei* phages, C3.1 and C4.1 (whose sequences were very similar), yielded 55 genes that encode proteins ranging from 30 to 1369 amino acids in length. Protein domains have been identified for 40 of these proteins, from which the putative function of individual ORFs can be determined. Among the genes observed were sequences encoding the small and large subunits of the terminase protein and the portal protein. The products of these genes are involved in the translocation and packaging of DNA into empty capsids. Several genes encoding structural proteins of the phage's capsid and tail were also present, as well as holin and endolysin, which are involved in the degradation of the bacterial host's cell membrane during the infection process. Also present was a gene encoding a site-specific integrase enzyme and a sequence that is likely to be necessary for the integration of phage genetic material into bacterial DNA. Comparative analysis showed that a similar *attP*/*attB* sequence is present in the genomes of several phages and bacteria of the *L. casei* group. Genes encoding proteins related to the transition from the lysogenic to the lytic cycle were also detected—genes encoding CI-like repressor, Cro-like repressor and phage antirepressor KilAC domain-containing proteins were present in the genomes of C3.1 and C4.1 phages. Numerous genes encoding proteins that contain domains responsible for interacting with nucleic acids have also been identified, including those that encode endonucleases or may be involved in the phage DNA replication process.

Two bacteriophages isolated from *L. paracasei* strains showed very little similarity to each other (92.7% sequence similarity, 2% query cover). Despite this, the identified genes (44 for phage P2.4 and 55 for phage P7.1) encoded functionally similar proteins both structural and also related to the folding of virus particles and lysis of bacterial host cell envelopes. Interestingly, the P2.4 phage sequence lacks the integrase gene, which may indicate the defective nature of this bacteriophage or may be due to incomplete assembly of the obtained sequence. In the case of phage P7.1, the gene encoding site-specific integrase, as well as the *attP* integration sequence, was located between the lysis fragment and a region of lesser-known function that contains, among other constituents, genes encoding: ImmA/IrrE family metalloendopeptidase, type I restriction enzyme and helix-turn-helix transcriptional regulators (Additional file 7).

Phage sequence analysis of strains belonging to *L. rhamnosus* revealed a mosaic nature of these genomes. This manifests itself in that a phage sequence could show great similarity to a previously described virus within several genes, while another fragment of the genome, despite similar gene function, could show no similarity to the same phage. The incredible variability of these bacteriophage sequences does not entail functional variability. That is, despite differing sequences within genomes, it is easy to identify genes with similar biological functions. In most cases, genomic sequences can be divided into the following functions: DNA packaging (small and large terminase subunit); the sequence encoding the structural proteins of the phage's capsid and tail; the lysis sequence, which usually consists of holin and lysine proteins; the region containing the gene encoding the enzyme integrase; and the sequence where recombination and fusion of phage and bacterial host genetic material takes place. Furthermore, present within regions in the genomes of the described phages are sequences related to the potential transition of the phage from the latent form to the lytic form (genetic switch) and also a fragment related to DNA replication. Bacteriophages R3.1, R3.3, R9.1, R10.1, R18.1, R23.9, R24.1, R24.2 and R29.1 have these features and a similar genome structure. These phages had genomes of about 38,000 to 44,000 bp, in which 54 to 67 protein-coding sequences were identified. In the case of R9.2 and R9.3 contigs, the comparative analysis showed that they are likely sequences of the same bacteriophage that could not be assembled into a complete genome. Within the R9.2 contig (16,700 bp, 30 ORFs), genes associated with phage integration, maintenance of lysogeny, and replication were detected. In contrast, no ORFs encoding structural proteins were observed, which in turn were identified within contig 9.3 (22,500 bp, 26 ORFs). In addition to genes encoding phage capsid and tail proteins, this contig contained gene sequences for the small and large subunits of the terminase. A similar situation occurred with the R26.1, R26.9, R26.14 and R26.26 contigs. In the genome of *L. rhamnosus* LMG 23551, for which these contigs were obtained, one prophage encoded by sequences present in contigs R26.1 and R26.14, and another in R26.9 and R26.26 were present (Table 4 and Additional file 7).

**Figure 3:**
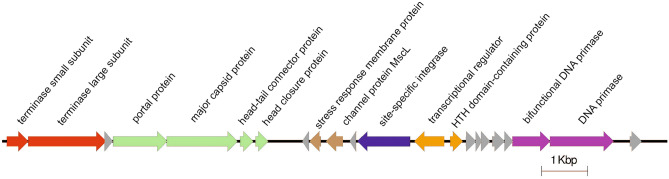
Schematic map of genome sequence determined for phage R23.1. Predicted functions of identified genes are labeled.

An interesting genome structure is shown by bacteriophage R23.1, which was isolated from a mitomycin C-induced culture of *L. rhamnosus* strain LMG 23277. The sequence is only 14,500 bp within which only 22 genes have been identified, so one might assume that it is only a fragment of the phage genome. Nevertheless, based on analysis of the genomes of bacteria belonging to the same species, it was possible to identify identical or very similar prophage sequences in 13 strains of *L. rhamnosus* (PMC203, IDCC 3201, hsryfm 1301, cek-R1, 1001311H_170123_H11, AMC0712, AMC0706, L3_133_000G1_dasL3_133_000G1_concoct_81, 708_LRHA, 699_LRHA, LRHMDP3, LRHMDP2 and L31). Of the 22 genes detected, the functions of as many as 9 were difficult to determine (hypothetical proteins) (Fig. 3). However, within the genome, we were able to identify genes encoding the large and small subunits of the terminase, as well as structural proteins, such as portal protein, major capsid protein and phage head closure protein. Noticeably absent are the sequences encoding typical phage tail proteins and the holin and lysine genes. Interestingly, sequences specific to bacterial membrane-associated proteins—GlsB/YeaQ/YmgE family stress response membrane protein and large-conductance mechanosensitive channel protein MscL—were present within the R23.1 phage genome. Learning the exact function of these proteins in this particular case requires further research. The ORFs of integrase, the gene related to maintaining a lysogenic state, and 2 genes related to the DNA replication process, were also detected in the genome (Fig. 3).

## Discussion

Research on bacteriophages that infect the *Lactobacillus* species of bacteria (which has now been divided into 25 genera) has focused mainly on lytic viruses that cause the lysis of bacterial cultures. This has its justification since contamination with such viruses can cause the inhibition of technological processes in which live microorganisms are used, therefore leading to substantial financial losses^22^. As the presence of lysogenic viruses does not cause such effects, it seems that knowledge of the occurrence and physiological importance of such bacteriophages, for both the bacterial host and the specific ecological niche is still insufficient. Owing to the development of bacterial genome sequencing technology, the scientific community now has access to an enormous number of bacterial genomic sequences, which has made it possible to determine that a significant percentage of genomic sequences are mobile fragments, including prophages. It is estimated that as much as 10–20% of bacterial genomic sequences may be sequences of phage origin. Up to a dozen prophage sequences have been detected in some bacterial strains, confirming the widespread distribution of such sequences in bacterial genomes^23–25^.

In the present study, we analyzed the occurrence of prophage sequences present in the genomes of the *L. casei* group, deposited in the NCBI database. More than 1500 such sequences were detected in nearly 440 genomic sequences, which were divided into 3 groups: intact, questionable and incomplete. Noteworthy is the large number of questionable and incomplete sequences; such results may be due to several factors. First, the limitations of the algorithm with which the analysis was performed. The division into groups based on a specific score itself seems to be a major simplification; nevertheless, regardless of the prophage sequence detection algorithm used, based on the results obtained, it can be unequivocally stated that phage sequences are widely distributed in the genomes of microorganisms belonging to the *L. casei* group. Another important issue seems to be the extreme variability of prophage sequences and the phenomenon of phage mosaicism, which can also make it difficult to detect such sequences and classify them as intact, fully functional prophages. The quality of assembly of the obtained bacterial genomic sequences also appears to be important. In the case of sequences deposited as contigs, sometimes single prophage sequences were detected within several short contigs, which precluded the classification of such sequences as intact. Finally, bacterial genomes often contain phage-derived sequences called cryptic phages that do not form fully active phage particles. It has been observed that such sequences can also significantly affect bacterial host metabolism^26–28^.

The present study analyzed 422 prophage sequences that scored at least 90 in the Phaster software. It is noteworthy that in the case of *L. paracasei* and *L. casei* strains, the phenomenon of polylysogeny was observed much more often compared to *L. rhamnosus* bacteria. Amongst other factors, this may be related to the greater homogeneity of the environment in which *L. casei* and *L. paracasei* microorganisms reside. The length of the analyzed sequences ranged between 7.7–74.4 kb. So far, the vast majority of bacteriophages previously described for the *L. casei* group had genomes of about 35–45 kb^29,30^. Of particular interest are the shortest sequences with the most reduced number of genes. Accurate characterization of such genomes, especially in the case of fully functional bacteriophages, may allow the determination of the minimum set of genes necessary to carry out the reproductive cycle and may be important in determining the direction of evolutionary changes that bacteriophages infecting a particular group of bacteria undergo. It is worth noting that within the described study, a bacteriophage (R23.1) was isolated that had a genome of only 14.5 kb in which 22 ORFs were detected. This bacteriophage showed no genetic similarity with bacteriophages previously described for *L. rhamnosus* species^5,13,31^. Of great interest is the enormous variation between the prophage sequences detected for the species studied. This is indicated, for example, by the average nucleotide identity, which was 32.7%. Prophage sequences appear to be the most genetically diverse elements of bacterial genomes. Therefore, these sequences are increasingly being considered useful for genotyping and identifying microorganisms at the strain level. The development of unique genetic markers specific to particular strains is important, both in epidemiological studies and in the precise identification of industrial strains whose use is covered by patent protection^32–34^.

Despite such a high diversity of sequences, the use of universal primers developed by Zaburlin et al.^7^ allowed the detection of prophage sequences in the genomes of nearly 90% of the tested strains of the *L. casei* group. Although in an earlier paper the authors divided the *L. casei* group phages into only 3 groups, which certainly does not reflect the genetic diversity of these sequences, the experimental set-up used confirms the wide distribution of prophages in the bacterial group studied. Based on the results, a group of strains was selected to obtain phage particles. The isolation source of the tested bacteria was also important—8 of the 12 strains were clinical isolates from patients with bacteremia and endocarditis. Numerous reports indicate that *Lactobacillus* can cause sepsis and other pathogenic conditions^35–37^. Some researchers suggest that prophage induction may be a factor that promotes bacterial host pathogenicity^38–40^. In this stage of the study, phage particles were successfully obtained from all selected strains using mitomycin C. Based on the genetic analysis of the phage material, it can be concluded that at least 1 phage sequence was detected for each of the strains tested. The results confirm previous conclusions obtained from bioinformatics analyses that *L. casei* group bacteria constitute a very rich reservoir of prophage sequences with extreme diversity. Causes of such variability in prophage sequences can include point mutations, transposon insertion, deletions as well as homologous recombination between 2 sequences located within a single cell^23,41^. It is worth noting here that enormous genetic variation must entail both structural and physiological variation. Hence, it can be assumed that the existing knowledge of bacteriophage biology, which is limited to a few types of reproductive processes, seems to be very limited.

The ecological aspect of the occurrence of prophage sequences in the microorganism genomes and the phenomenon of spontaneous induction of prophages are of particular interest. Numerous studies indicate that, in addition to classical lysis, prophage sequences can undergo gradual spontaneous induction as a result of various extracellular and/or intracellular factors^42^. It has been shown that during culture under laboratory conditions, there is a several-fold increase in the number of free bacteriophages^5,12^. At this point, it is also worth highlighting the multilevel importance of lysogeny and spontaneous prophage induction. Studies have shown that lysogenic phages not only affect the physiology of bacterial host cells but can also play an important role in shaping the entire bacterial community within an ecological niche^2,43,44^. When prophages are carried by intestinal bacteria, it can be assumed that by influencing the composition of the intestinal microbiota, prophages indirectly affect the physiology of the gastrointestinal tract, and thus the overall well-being of humans^21,45,46^.

By analyzing the importance of prophages for the bacterial host, it can be concluded that their presence in bacterial genomes influences the genetic diversity of microorganisms and may regulate the expression of certain genes. Prophages can also give bacteria an advantage in a given ecological niche by introducing various traits—pathogenicity factors or resistance to other bacteriophages, for example. Moreover, it has been proven that spontaneous prophage induction promotes biofilm formation, and can indirectly promote bacterial virulence as well as being an important phenomenon contributing to horizontal gene transfer^42,43,47,48^. Thus, SPI may be the key process by which genetic variation is built upon, not only in bacteria but also in bacteriophages themselves. It is worth noting that despite the widespread prevalence of lysogeny in bacteria of the genus *Lactobacillus*, only a few studies have focused on the physiological significance of the presence of prophages in the genomes of these bacteria. Therefore, further research involving not only the characteristics of the new viruses themselves but also the importance of these viruses from an industrial, medical or ecological point of view, is essential to explore the application potential of bacteriophages, as well as to understand their significance from the perspective of how entire ecosystems function.

## Methods

### Prophage identification in genomes of *L. casei* group

Nucleotide sequences of 5 bacterial species—*Lacticaseibacillus casei*, *Lacticaseibacillus paracasei*, *Lacticaseibacillus zeae, Lacticaseibacillus rhamnosus* and *Lacticaseibacillus chiayiensis*—were downloaded from the NCBI database. Files with a total of 439 sequences were obtained, of which 37 contained full bacterial genomes and 402 contig sequences. Downloaded data were analyzed with the Phaster program^26^. The obtained phage sequences (intact type) were then subjected to comparative analysis using Kalign 3 software^49^. The following parameters were used for analysis: gap open penalty: 11.0; gap extension penalty: 0.85; and terminal gap extension penalty: 0.45. The sequence alignment was calculated using the ClustalO program^50^. Comparative analysis of full genomes, presented using a dot plot, was performed with the Gepard software^51^. Intact nucleotide sequences underwent clustering with a 45% identity threshold^19^. Clusters were created with the assumption that there must be at least 3 sequences in a single cluster. Clustering was performed using the MMseqs2 software^52^.

### Microorganisms and bacterial culture conditions

In the next part of the study, 56 strains of *L. casei* group bacteria were used from two international collections of microorganisms—Japan Collection of Microorganisms (JCM) and Belgian Coordinated Collections of Microorganisms (BCCM) (Table 3). Bacteria were cultured in MRS medium at 30 °C or 37 °C. The growth dynamics of bacterial strains in the presence of various concentrations of mitomycin C were observed using a Bioscreen C device (OY Growth Curves).

### Polymerase chain reaction conditions

Bacterial DNA samples were prepared using the commercial Genomic Mini AX BACTERIA + kit (A&A Biotechnology). Polymerase chain reaction (PCR) reactions were conducted in a T100 thermocycler (Biorad) using primer sets previously described by Jarocki et al. for bacterial species identification, and by Zaburlin et al. for prophage detection^7,20^. Electrophoretic separations of DNA were conducted in a SubCell Mini-Sub Cell GT electrophoresis device (Biorad). The results were analyzed using Gel Doc XR+ gel documentation system (Biorad).

### Bacteriophage particles isolation

MRS media (Oxoid) supplemented with 10 mM CaCl_2_ was inoculated with 2 ml of fresh bacterial culture (1:100). After 4–5 h at an OD600 of 0.2–0.5, 200 µl of mitomycin C (final concentration of 0.5 µg/ml) was added to the culture. The culture was kept at 37 °C for 18–20 h. After incubation, the culture was centrifuged for 10 min at 5000×*g*, at 4 °C. After the first centrifugation was completed, the supernatant was carefully decanted into a clean tube and centrifuged again under the same conditions. Next, NaCl and PEG 8000 (Merck) were added to a flask of culture fluid, with final concentrations of 0.5 M and 10% w/v, respectively. The flask was incubated at 4 °C, on a magnetic stirrer. After about 20 h, the culture fluid was centrifuged for 1 h at 18,000×*g*, at 4 °C. The supernatant was removed while the pellet was centrifuged again for 5 min under the same conditions. The residual supernatant was then removed, and the pellet dissolved in 2–4 ml of SM buffer. The samples were stored at − 20 °C.

### Sequencing and genome characterization of selected bacteriophages

Phage DNA was prepared using Phage DNA Isolation Kit (Norgen Biotek) according to the manufacturer’s instructions. The concentration of genomic DNA was measured before the library preparation procedure by fluorimetry using PicoGreen reagent (Life Technologies). The measurement was performed on the Infinite M200 instrument from Tecan. Genomic DNA was fragmented by sonication using the Covaris E210 instrument (Covaris), according to the parameters recommended for preparing libraries for Illumina sequencing. The libraries were prepared using the NEBNext^®^ Ultra™ II DNA Library Prep Kit for Illumina^®^ (New England Biolabs) according to the manufacturer's recommendations. Sequencing was performed with the MiSeq sequencer, in paired-end (PE) mode, 2 × 300 bp, using kit v3 (Illumina), according to Illumina's protocol. The raw readings were subjected to quality control and filtered accordingly. All readings meeting the quality requirements were submitted de novo to the CLC Genomic Workbench (CLCBio) program. The beginning and end of the phage genome were determined using PhageTerm^53^ where reads paired in the CLC program were re-mapped to the phage contig. The tool's task was to determine the starting position coverage (SPC) and confront it with the coverage in each orientation of the DNA molecule. In cases where the program was unable to determine the ends, the sequence obtained in the CLC Genomic Workbench program was used for further analysis. Annotations were conducted independently using the PROKKA platform (based on a viral database), using PRODIGAL software to identify open reading frames (ORFs)^54,55^.

## Supplementary Information

Supplementary Information 1.

Supplementary Information 2.

Supplementary Information 3.

Supplementary Information 4.

Supplementary Information 5.

Supplementary Information 6.

Supplementary Information 7.

## Data Availability

The phage genome sequences have been deposited in the GenBank database under accession numbers: OP676228, OP807341–OP807346, OP869843–OP869855. All data generated or analyzed in this study are included in the article and its supplementary information files.
